# Regional health care planning: a methodology to cluster facilities using community utilization patterns

**DOI:** 10.1186/1472-6963-13-333

**Published:** 2013-08-22

**Authors:** Paul L Delamater, Ashton M Shortridge, Joseph P Messina

**Affiliations:** 1 Department of Geography, Michigan State University, East Lansing, MI 48824, USA; 2 Center for Global Change and Earth Observations, Michigan State University, East Lansing, MI 48824, USA; 3 Michigan AgBioResearch, Michigan State University, East Lansing, MI 48824, USA

**Keywords:** Health care utilization, Hospital planning, Certificate of need, Clustering, K-means, Ward’s

## Abstract

**Background:**

Community-based health care planning and regulation necessitates grouping facilities and areal units into regions of similar health care use. Limited research has explored the methodologies used in creating these regions. We offer a new methodology that clusters facilities based on similarities in patient utilization patterns and geographic location. Our case study focused on Hospital Groups in Michigan, the allocation units used for predicting future inpatient hospital bed demand in the state’s Bed Need Methodology. The scientific, practical, and political concerns that were considered throughout the formulation and development of the methodology are detailed.

**Methods:**

The clustering methodology employs a 2-step K-means + Ward’s clustering algorithm to group hospitals. The final number of clusters is selected using a heuristic that integrates both a statistical-based measure of cluster fit and characteristics of the resulting Hospital Groups.

**Results:**

Using recent hospital utilization data, the clustering methodology identified 33 Hospital Groups in Michigan.

**Conclusions:**

Despite being developed within the politically charged climate of Certificate of Need regulation, we have provided an objective, replicable, and sustainable methodology to create Hospital Groups. Because the methodology is built upon theoretically sound principles of clustering analysis and health care service utilization, it is highly transferable across applications and suitable for grouping facilities or areal units.

## Background

Health care planning and regulation in the United States has generally attempted to achieve two broad goals: 1) promote public health by ensuring that the supply of services meets the population’s need and 2) contain health care costs by regulating the supply of services to a level congruent with the need of the population. Regulation is often enforced through state-level Certificate of Need (CON) programs, which attempt to enable a sufficient supply of service to meet the population’s health care needs without providing a large oversupply or duplication of services [1]. CON programs require that proposals for additional health care services or facilities demonstrate an unmet need prior to approval. Although their merits have been questioned over the past 40 years (see [1-3]) and they are no longer federally mandated, 36 states in the US currently employ some form of CON program [4].

Health care services are used by people, but are supplied by health care professionals who deliver these services at hospitals, clinics, and other facilities. Although the demand for hospital services can be considered an attribute of people or populations, the supply only exists at hospitals. Further, the areal units used to aggregate populations rarely, if ever, contain residents who use a single health care facility [5]. In an effort to enable community-based planning of health care resources, communities and/or hospitals are grouped to form regions of similar health care use. Thus, planning occurs at a regional level wherein the supply of health care resources available to the larger community are measured against the community’s need. In the US, 28 CON states predict or evaluate the relationship between hospital bed supply and demand [4], necessitating methods or techniques for grouping both population units and hospitals (e.g., [6-8]).

Very limited research emphasis has been placed on grouping or clustering hospitals based on similarity in community utilization patterns. Methods for clustering hospitals using multivariate data received attention from health services researchers in the 1970s and 1980s. These studies, however, were more focused on identifying hierarchical structure in the overall system of hospitals or identifying similarity among hospitals for determining reimbursement levels [9-13]. More recently, this research topic has been revived in response to changes in health care delivery and organization [14-16].

A review of the literature provides little guidance toward alternative or improved methods to group health care facilities. The branch of research most related to this particular problem is the creation of small areas. Yet, the methods used to create small areas have also received little attention [17]. Although multiple methods have been proposed more recently to group communities into health service regions (e.g., [18-20]), they are extensions of the straightforward, yet unsophisticated, plurality method employed by Wennberg and the Dartmouth Atlas group [21].

Here, we present a new clustering methodology that groups hospitals based on similarity among their overall pattern of community utilization and geographic location. Despite being developed within the politically-charged atmosphere surrounding CON regulation, the methodology offers an objective, replicable, and sustainable solution for grouping hospitals. Importantly, the methodology uses generally accepted clustering techniques and can be easily transferred to group other types of health care facilities or to create small areas for health service studies. Further, the source code necessary to replicate our clustering methodology is provided to ensure that the specific techniques we employ are unambiguous.

## Clustering

The overall objective in most clustering analyses is to assign individual observations into natural groups or clusters. Jain ( [22]p. 652) states that the operational definition of clustering is: 

Given a *representation* of *n* objects, find *K* groups based on a measure of *similarity* such that the similarities between objects in the same group are high while the similarities between objects in different groups are low.

A large majority of clustering algorithms can be described as either hierarchical or partitional in nature. Hierarchical algorithms use an *n* × *n* similarity matrix to recursively form nested clusters over all possible values of *K*. Partitional algorithms divide observations into a user-defined number of clusters and utilize an *n* × *n* similarity matrix or an *n* × *m* matrix of observations, where *n* observations have *m* attributes or data dimensions.

Applied cluster analysis requires the analyst to make a number of subjective decisions. Prior to clustering, the attributes (or variables) used to describe similarity among observations must be determined, a potentially subjective process [11]. Additionally, a large number of clustering techniques exist, creating a “user’s dilemma” in the technique selection process [23]. Finally, determining the number of clusters or groups, *K*, is one of the most difficult problems in cluster analysis [22,24]. Milligan and Cooper [25] provide a comprehensive review of clustering and cluster analysis, offering a seven-step structure to guide the clustering process: 1) select the entities, 2) select attributes of similarity, 3) decision on data standardization, 4) select similarity measure, 5) select a clustering algorithm, 6) determine the number of clusters, and 7) evaluate and interpret the output clusters.

## Case study

Our case study was conducted in 2011, during a substantial review of the methodologies contained within Michigan’s Hospital Bed (HB) Standards (see [26]). These Standards carry the power of law [27] and are used by the state’s CON Program to regulate the availability of hospital beds. In the HB Standards, the specific methodologies employed to define Subareas and calculate the necessary supply of hospital beds needed to meet the state’s future population demand (Bed Need Methodology) are detailed.

**Figure 1:**
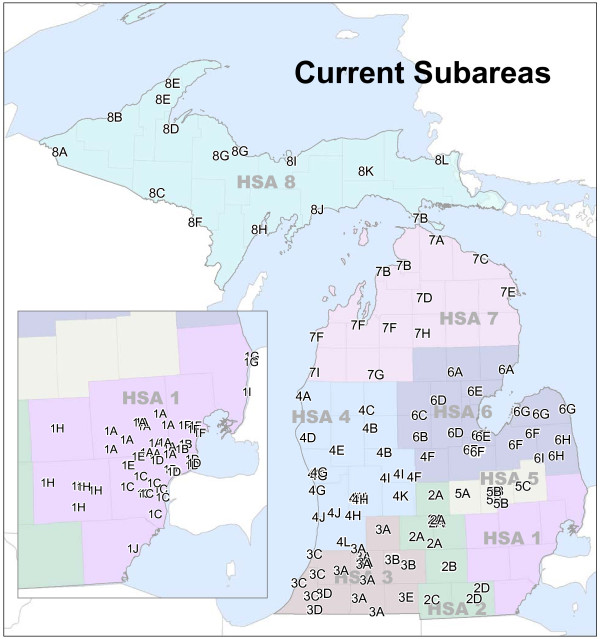
**Michigan’s current subareas.** Labels indicate hospital location and Subarea membership. Underlying colors represent Michigan’s Health Service Areas (HSAs).

The state’s Subareas are the units of allocation used within the Bed Need Methodology (see Figure 1). John Griffith, J. William Thomas, and colleagues explored the subject of identifying health-based service communities over 30 years ago [28-31], providing a methodology that clusters communities and hospitals simultaneously. The State of Michigan adopted the Thomas methodology [30] for the creation of Michigan’s Subareas.

Michigan’s CON Commission assembled a Hospital Bed Standard Advisory Committee (HBSAC) in 2011 to explore potential modifications of the Bed Need Methodology. The HBSAC, in turn, formed a working group that was composed of various stakeholders in Michigan’s health care industry and was tasked with conducting a critical evaluation of the Thomas clustering methodology and providing alternative approaches if deemed necessary. The study authors participated in the HBSAC working group, providing scientific support throughout the process. In the following paragraphs, we detail a number of the practical and scientific concerns raised within the working group regarding the Thomas Methodology and the state’s Subareas. Although these concerns focus the Thomas Methodology and Michigan’s Subareas, they highlight the necessity to explore alternative approaches for clustering hospitals and the context in which our new methodology was developed.

**Figure 2:**
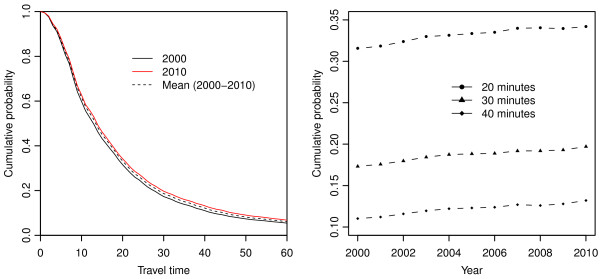
**Changes in travel time behavior for hospitalizations (2000-2010).** (L) Travel time is the number of minutes between the patient’s residence and the hospital. Cumulative probability is the proportion of patient days utilized at hospitals greater than or equal to the specific travel time. For example, the cumulative probability at 0 minutes is equal to 1 because all hospitalizations occurred at a hospital located 0 minutes or more from the patient’s residence. In another example, the percentage of patient days at hospitals 30 minutes or further from the patient’s residence is roughly 20% in 2010 and roughly 17.5% in 2000. (R) Yearly values of cumulative probability for 20, 30, and 40 minutes are shown.

The first broad concern was in regard to the overall legitimacy of the Subarea configuration. Both a practical perspective (e.g., do the Subareas reflect current use patterns?) and theoretical perspective (e.g., do the Subareas reflect current trends in hospitalization use and travel behavior?) were considered. Despite efforts to trace the history of Michigan’s Subareas, detailed records or verifiable accounts of previous configurations were unable to be located. Outside of minor changes in 2002, we believe that the Subarea configuration had not undergone modification since the original formulation in the late-1970s. Moreover, many Subareas consisted of only a single hospital (of the 64 Subareas in Michigan, 32 were “single hospital Subareas”). Despite known changes occurring in the acute care hospital system in the US (e.g., consolidation [32], patent targeting [33], and rural hospital bypass behavior [34]) and those occurring in the specific utilization patterns and travel behavior of Michigan’s communities over the past 30–40 years, the Subarea configuration remained relatively constant. Figure 2 illustrates that travel time for hospitalizations in Michigan has gradually increased from 2000–2010.

Re-implementing the Thomas methodology, which includes an initial automated clustering method and a secondary step where the results are reviewed and modified by an expert panel, was also found to be problematic. Although a detailed description of the methodology is offered in Thomas et al. [30] and the Hospital Bed Standards [26], portions of the methodology remain cryptic. A similar problem was experienced by researchers at Michigan State University when tasked with implementing Michigan’s Bed Need Methodology (detailed in Langley et al. [35]). Therefore, the initial action required explicitly defining the Thomas Methodology (see Additional file 1) and implementing the methodology with up-to-date population and hospital utilization data. We used the R programming language and environment [36] to complete this task. However, only the clustering algorithm was implemented, given the lack of information regarding the scope and nature of modifications made to the Subareas via the expert panel.

**Figure 3:**
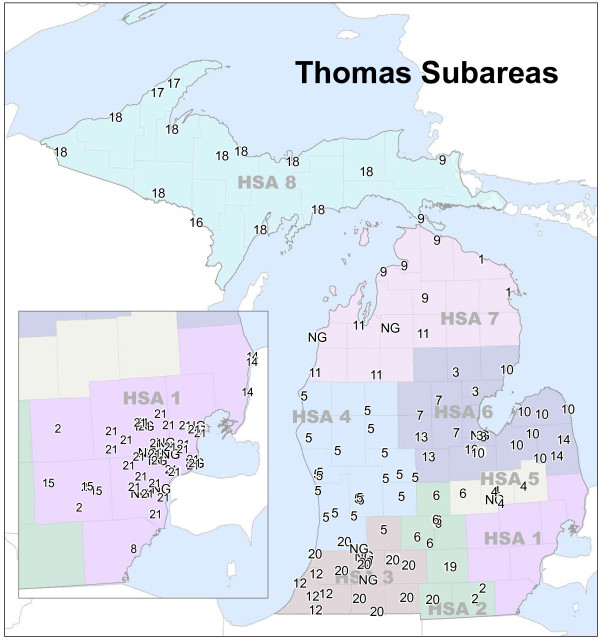
**Subareas produced by the Thomas Methodology using current data.** 34 hospitals did not possess the required minimum home area to be included in the Thomas Methodology. Because no details are provided by the methodology with regards to handling these cases, they were removed from the clustering process. They have been assigned NG (non-groupable) for display purposes.

The final concern was the suitability of the Thomas methodology for grouping hospitals, given changes in health care access and delivery in the 30 years since its adoption. As Figure 3 illustrates, the Thomas Methodology does not provide a solution resembling the current Subarea configuration when implemented with recent hospitalization data^a^. Most notably, only 21 Subareas were identified. The dissimilarity is likely attributable to state-wide changes in hospital utilization behavior and utilization patterns occurring since the last time the methodology was implemented.

The practical concerns identified in the Thomas Methodology induced a further exploration of the theoretical underpinnings of the methodology, including a robust analysis of the input data and overall approach to clustering. These are presented and discussed further in an effort to highlight the complex set of factors that are likely to arise in any clustering application (as detailed by Milligan and Cooper [25]). We focus on hospitals and the Thomas Methodology in particular; yet, the concepts presented are generalizable such that they are applicable for grouping any type of observations having geographic utilization data for attributes.

### Hospital similarity

The Thomas Methodology clusters hospitals based on overlapping home areal units, defined by patient utilization patterns expressed using Relevance Index (*RI*) values. For a hospital *i* and a population unit *j*, the pairwise *RI* value is defined as

(1) $${\text{RI}}_{i,j}=\frac{{\text{Pd}}_{i,j}}{{\text{Pd}}_{j}},$$

where *Pd*_*i*,*j*_ is the number patient days used by residents of areal unit *j* at hospital *i* and *Pd*_*j*_ is the total number of patient days used by residents of areal unit *j* (at all hospitals) [28]. An *RI* value can be calculated for every hospital and areal unit pair under consideration to form a *RI* matrix.

The set of *RI* values for a single hospital characterize the “importance” of that hospital to each of the areal units. Because the patient days in the denominator of Eq. 1 are the total of the areal unit, the measure is highly influenced by the size of the hospital (number of beds). Further, *RI* provides more pertinent information about the community’s utilization trends, rather than the hospital’s. For example, if 75% of population unit *j*’s hospital usage is supplied by hospital (*RI*_*i*,*j*_ = 0.75), it is obvious that hospital *i* is very important to population unit *j*. However, what this value does not provide is information regarding the magnitude of population unit *j*’s contribution to hospital *i*’s overall patient distribution.

The Commitment Index (*CI*) provides an alternative representation of patient utilization patterns. A hospital-centric approach, *CI* values measure the importance of each population unit to the hospital. *CI* is defined for a hospital *i* at each population unit *j* such that

(2) $${\text{CI}}_{i,j}=\frac{{\text{Pd}}_{i,j}}{{\text{Pd}}_{i}}$$

where *Pd*_*i*,*j*_ is the number patient days used by residents of areal unit *j* at hospital *i* and *Pd*_*i*_ is the total number of patient days at hospital *i* (from all areal units) [28]. Unlike *RI*, *CI* values are not overly influenced by the size of the hospital. As a result, *CI* values provide a characterization of utilization patterns that are directly comparable among hospitals; further, past research has employed *CI*-based utilization patterns to identify hospital-based geographic service areas (e.g., [37-39]).

**Figure 4:**
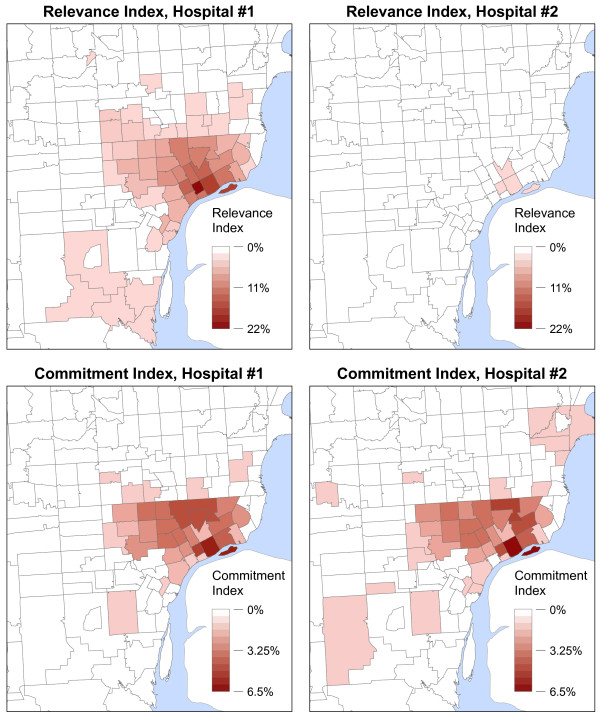
***RI *and *CI* maps for two hospitals of different sizes.** Hospital #1 has roughly 5 times more licensed inpatient beds than Hospital #2. The hospitals are located within one mile from one another. The classification schemes for *RI* and *CI* values are held constant between maps for comparative purposes.

**Figure 5:**
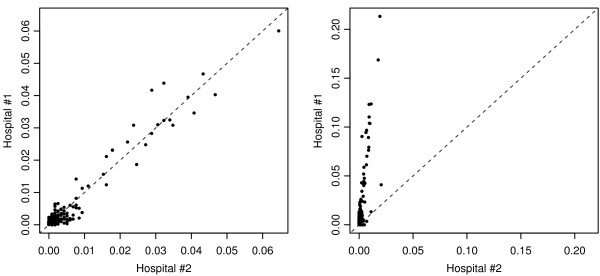
***RI ***and ***CI *values two hospitals of different sizes.** CI values (L) and *RI* values (R) are plotted for the two hospitals across areal units (e.g., Hospital #1’s *RI* in Areal Unit #1 plotted against Hospital #2’s *RI* in Areal Unit #1). A 1:1 line has been added for reference.

Figure 4 illustrates how *RI* and *CI* may provide significantly different representations of patient utilization patterns. In the example, two hospitals are located very near each other, with one having a large number of beds (#1) and one having a lesser number (#2). The maps clearly show that the *RI* values vary greatly between the two hospitals; however, the *CI* values of the two hospitals appear to be quite similar. Figure 5 shows the *CI* and *RI* values for the two example hospitals plotted against each other.

*RI* and *CI* both provide salient information about hospital utilization patterns. Yet, the differences are apparent when attempting to characterize the geographic distribution of a hospital’s patient base for comparative purposes. As Figures 4 and 5 illustrate, the two example hospitals draw nearly the same proportions of their overall patient population from the exact same areal units (the hospitals’ *CI* values closely follow the 1:1 line); specifically, the hospitals have highly similar geographic footprints within the region. The Pearson’s correlation coefficient (*r*) of the hospitals’ *CI* values confirms the similarity, showing nearly perfect correlation (*r* = 0.975). Although correlation is also high between the hospitals’ *RI* values (*r* = 0.855), the similarity in community utilization patterns is not nearly as apparent due to the differences in magnitude of the *RI* values (which are heavily dependent upon the number of beds at each facility). The RMSE (Root Mean Squared Error) between the hospitals’ sets of *CI* values (0.0018) is an order of magnitude less than RMSE of the *RI* values (0.0227), confirming these large differences.

### Clustering approach

In the Thomas Methodology, clusters are formed via an evaluation of the proportion of patient visits originating in “home areal units” (expressed as *RI* values). A hospital’s home areal unit is assumed to be the geographic region in which the hospital is located^a^. The approach to clustering can be simplified as such: if Hospital A (located in Unit A) provides a small proportion of the hospitalizations for Unit A (it has a low *RI* value), Hospital A will be grouped with the hospital having the highest *RI* value in Unit A. This process continues iteratively, in a watershed-type fashion, until specific user-supplied thresholds are reached.

Three major theoretical shortcomings permeate this approach to clustering hospitals. The first is the assumption that similarity among hospitals can be adequately assessed based on *RI* values in a single areal unit. As Figure 4 demonstrates, a hospital’s patient base is highly likely to be distributed throughout a region and not confined to a single areal unit. Considering whether to cluster hospitals based solely on the overlap in a single areal unit largely ignores where the “rest” of the hospitals’ patients originate. Specifically, the overall geographic distribution of hospital utilization is not evaluated in this procedure; similarity among hospitals is reduced to an evaluation of whether one hospital draws a specific (user-defined) proportion of patients from the home areal unit of another hospital.

The Thomas Methodology’s definition of home areal units is the second theoretical shortcoming. The methodology assigns a single home areal unit for a “group” of hospitals once they have been clustered. Specifically, the home areal unit of the entire cluster is designated as the home areal unit of a single cluster member hospital. Because the methodology further clusters these groups based on overlap within this single home areal unit, it does not acknowledge that the cluster contains multiple hospitals. This has the potential to result in scenarios where hospitals grouped into the same Subarea may share little to no similarity. For example: 

• Hospital A is clustered with Hospital B based on Hospital B’s *RI* in Hospital A’s home areal unit.

• Once the hospitals are clustered to form Cluster AB, the home areal unit is assigned as Hospital B’s home areal unit.

• When Cluster AB is further clustered with Hospital C, the criteria for clustering is based on Hospital C’s *RI* value in Cluster AB’s home areal unit. Because Cluster AB’s home areal unit is defined as Hospital B’s home areal unit alone, utilization within Hospital A’s home areal unit is not considered.

In this scenario, Hospital C and Hospital A may share little or no similarity in the newly formed Cluster ABC. Because the Thomas Methodology iterates until there is little overlap among home areal units, very large clusters (see Clusters #5 and #18 in Figure 3) or geographically discontinuous clusters (see Cluster #10 in Figure 3) may be created such that patient utilization patterns for hospitals on the periphery are dissimilar to those of the other cluster members.

The third theoretical shortcoming of the methodology is the use of subjective parameters (λ values for both single hospitals and hospital clusters) as the threshold level for determining whether hospitals should be clustered and as a “stopping point” of the clustering algorithm. Notably, in Thomas et al. [30], the authors state that the specific λ parameters were determined by running the algorithm with varying values until an acceptable cluster solution was reached. This method of parameter estimation is largely subjective, relying solely on the analyst’s interpretation of the appropriateness of the output clusters. As a result, the specific parameters are highly value-laden, without any general theoretical foundation or empirical justification for support.

### Subjective modification by expert panel

In the Thomas Methodology, the Subareas provided by the automated clustering algorithm are passed along to an expert panel for modification. Thomas et al. ( [30]p. 46) state: 

Based on members’ knowledge of hospital relationships and other factors influencing the reasonableness of proposed groupings, the committee is asked to decide whether the objectively determined clusters are in fact appropriate.... Thus the committee makes the final determination, using the patient origin data analysis as one important source of information.

Although this step offers the potential to incorporate useful qualitative or local knowledge into Subarea formulation, it also raises practical and theoretical concerns with regards to implementation. Allowing the Subareas to be modified *post hoc* adds another layer of subjectivity to the overall methodology, also providing an opportunity for the results to be “gerrymandered”, an unfortunate but important consideration in CON-related proceedings.

## Methods

Our overall goal in creating the new methodology was that it employed methods and procedures that are as ***objective***, ***replicable***, and ***sustainable*** as possible. Considering the subjectivity present in many clustering applications and the vast number of possible clustering methods, we placed emphasis on examining higher-level theoretical issues, rather than specific application-oriented concerns. Milligan and Cooper’s 7-step structure for clustering was used for guidance throughout the process.

Preliminary work focused on the identification of measurable hospital characteristics that could be used to compare and cluster similar hospitals. Two characteristics were deemed as the most important, as they are directly related to the final use of the clusters within the Bed Need Methodology: 1) that hospitals draw their patients from a similar set communities and 2) that hospitals are geographically proximate.

Furthermore, the term *Subarea* was replaced with *Hospital Group* in the new methodology to better reflect the nature of and specific use of the units within the context of the overall Bed Need Methodology. Specifically, the use of Subarea as the term to describe a collection of hospitals erroneously denotes that the group represents an “areal” feature.

### Overview of the new methodology

The new clustering methodology employs a 2-step K-means + Ward’s algorithm to create Michigan’s Hospital Groups. This algorithm compares observations across multiple attribute values, allowing for both community utilization patterns and hospital location to be evaluated simultaneously during cluster formation. Hence, the output clusters are groups of hospitals that 1) draw a similar proportion of their patient days from a similar set of communities and 2) are located near each other. Georeferenced hospitalization data and travel distance measurements among hospitals are required to implement the methodology.

The methodology also includes a heuristic to determine the number of Hospital Groups, *K*, based on statistical measures of cluster fit and characteristics of the Hospital Group solution. Further, we include a set of techniques to assign a *new* or *proposed* hospital to the existing Hospital Group solution in case this scenario arises.

The source code used to implement the overall methodology can be found in Additional file 2^b^. We utilize the R programming language using only base package functions to allow for portability across operating systems. The code has also been modified slightly from the actual code used for Michigan in an effort to make it more generalizable and transferable across applications. In the following sections, we provide a detailed description of the clustering approach and the data and procedures required to implement the clustering methodology.

### Clustering approach and attributes of similarity

The approach used in the new clustering methodology departs substantially from previous approaches for grouping hospitals and other health care facilities. Most notably, the new methodology does not require the delineation of clearly defined “service” or “market” areas for each facility or set of facilities. Service areas are required in these approaches as they employ some form of threshold value for clustering that is based on the overlap or market penetration within the areas. For example, the Thomas Methodology uses overlap within the home areal unit (defined by the *RI* values and λ parameter) to determine whether to cluster facilities (a similar approach is suggested in [37]). Other approaches (see [39]) choose a specific number of overlapping areal units among service areas to determine whether facilities should be clustered.

We do not consider a user-defined threshold of overlap within hospitals’ user-defined service areas to be a sufficient characterization of hospital similarity. On a conceptual level, both service areas and thresholds that determine a “groupable” amount of similarity are difficult to justify, given the subjective and arbitrary nature. Our new clustering methodology provides a more objective approach, by considering utilization throughout the entire study domain. To accomplish this, hospital similarity is based upon the overall geographic distribution of hospital’s total patient day population, represented by *CI* values. Because *CI* values are not overly sensitive to the size of a hospital, they provide a suitable metric for comparing any hospital under consideration. Further, by using the 2-step K-means + Ward’s algorithm, the new methodology is built upon a well-understand and accepted procedure to identify clusters when observations have multiple attribute values and similarity across all attributes is required.

### Input data

The methodology requires georeferenced hospital utilization data. We employ data from the Michigan Inpatient Database (MIDB), a nearly exhaustive record of the state’s inpatient hospitalizations. Each patient record includes the discharging hospital, the zip code of the patient’s residence, patient demographic information, and diagnostic codes. Using the most recent three years of MIDB data, the number of patient days used at each hospital by residents of each Michigan zip code are arranged in an *n* × *z* origin-destination (OD) matrix. Three years of data are included to ensure that recent patterns of state-wide hospital utilization are captured without the fluctuations possible in a single year. All existing hospitals that reported their inpatient data to the MIDB for any portion of the three year period are included. In this, reporting is essentially universal throughout the state’s hospitals. The *n* × *z* matrix of patient days is converted to a *CI* matrix (for each hospital in *n*) using Eq. 2.

The geographic location of each hospital is represented as an 1 × *n* vector of the travel distances to the other hospitals in the state. When consolidated, this results in an *n* × *n* OD distance matrix. The use of an *n*-dimensional representation of location, in lieu of traditional 2-D locational attributes such as x,y geographic coordinates, is necessary to account for Michigan’s particular physical characteristics and transportation infrastructure. Most notably, Euclidean distance measurements may lead to misrepresentations of true distances among locations near shorelines. For example, using only x,y coordinates to define location, hospitals in Michigan’s “thumb” region (HSA 6) in Figure 1 would be considered *near* hospitals to their northwest, not accounting for the true magnitude of their land separation due to the Saginaw Bay. Distances among hospitals are calculated as travel distances on Michigan roads using a custom-built network model [40]. After the *n* × *n* matrix is assembled, the distance entries are rescaled from 0 to 1 by dividing each by the maximum distance between any two hospitals. The rescaling process ensures that the range of values in the hospital utilization matrix and distance data matrix are similar [41,42].

The utilization matrix and distance matrix are joined to form a final data matrix containing *n* rows or observations with *m* (*z* + *n*) attribute values per observation.

### Clustering algorithm

The K-means clustering algorithm is employed as the primary method to create Hospital Groups. The specific algorithm employed is that of Hartigan and Wong [43], the default option in R’s base package kmeans() function. Given a set of *n* observations with *m* associated attribute values to be partitioned into *K* clusters, K-means attempts to find the cluster solution (*C*) that minimizes the sum of the squared errors (*J*(*C*)) between cluster members (*x*_*i*_) and their associated cluster center (*c*_*k*_) over all clusters.

(3) $$J(C)=\overset{K}{\underset{k=1}{\sum }}\underset{x_{i}\in c_{k}}{\sum }∥x_{i}-\mu_{k}{∥}^{2}$$

Therefore, given the input data for this application, the K-means algorithm attempts to minimize differences across *m* dimensions, evaluating the hospitals’ patient utilization patterns and geographic location simultaneously. The cluster centers produced by the algorithm represent the “central” location (in *m* dimensional space) of the cluster members’ hospitals. By minimizing *J*(*C*), the algorithm assigns hospitals having similar overall *CI* values and locational attributes into the same cluster, while also maximizing the “differences” between clusters.

Two distinct characteristics of the basic K-means solution provided concern for identifying Hospital Groups. First, solving Eq. 3 is an NP-hard problem [44], essentially rendering it non-computable for large problems in any acceptable amount of time. Thus, K-means implementations rely on a search algorithm to approximate the solution and likely provide locally optimal solutions, rather than the globally optimal solution [22,45]. Second, due to the first concern, the basic K-means method employs a random initialization procedure for the search algorithm. Given that the input data were of high dimensionality and complexity, the resulting Hospital Group solution identified by the randomly initiated K-means algorithm would likely vary slightly between model runs. Therefore, the results would not be reproducible.

To examine the variability associated with the random initialization of K-means and for the presence of local optima, we initially grouped the hospitals into 50 clusters using 5,000 random starting locations. Although there were roughly 9 × 10^203^ possible solutions^c^, the observed variability in the output cluster solutions was much higher than initially expected; each random start provided a unique 50 cluster solution.

**Figure 6:**
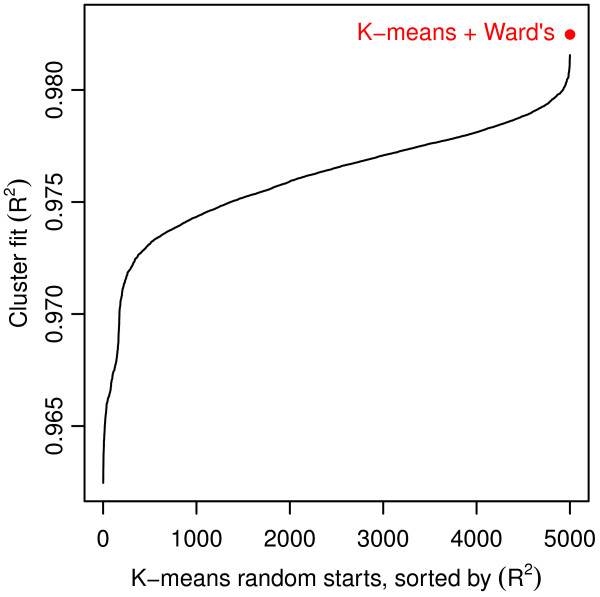
Local minima and random starting locations with the K-means algorithm. 5,000 K-means model runs for K = 50 produced 5,000 unique cluster solutions (black line). A single model run of the K-means + Ward’s algorithm provided another unique cluster solution with a better fit (red point) than any of the 5,000 stand-alone K-means solutions.

To stabilize the clusters provided by the K-means algorithm, we “seed” it with rational starting locations in lieu using of the random start method [46]. Ward’s hierarchical clustering algorithm [47] was employed to initially cluster the hospitals and provide the seed locations. The cluster centers produced by Ward’s algorithm are a *K* × *m* set of locations that define the central location of each cluster in *m*-dimensional space. They are used as initial locations in the K-means search algorithm, creating a 2-step K-means + Ward’s clustering algorithm. Because Ward’s algorithm provides deterministic results, this effectively and efficiently removed the stochasticity present in K-means initialization. In addition, for *K* = 50, the cluster solution identified by K-means + Ward’s provided a superior fit compared with *all* solutions from the 5,000 model runs using K-means with random starts (see Figure 6). Although we cannot confirm that the K-means + Ward’s algorithm provided the globally optimal solution, we are encouraged that a single model run produced such a large improvement in the fit of the cluster solution.

### Determining the number of hospital groups

As was discussed earlier, one of the more difficult problems facing any applied cluster analysis is determining the number of clusters in which to group the data. Researchers have noted that the selection of *K* is largely subjective [10], may be politically influenced [11], or completed by an analyst with expert domain knowledge [22] (as noted in our discussion of the Thomas Methodology).

The HBSAC working group requested that the number of Hospital Groups (*K*) be derived from the data itself, not explicitly predetermined prior to the clustering process nor modified after clustering is completed. However, no method or measure exists that is able to definitively answer the question, “how many clusters should the data be grouped into?”. Therefore, to complete this task in our methodology, we developed a heuristic that determines the number of Hospital Groups, integrating a statistical approach and a set of user-defined decision rules.

We define *k* as the set of integer values from 2 to *n*-1. A Hospital Group solution is created for each value in k using the K-means + Ward’s clustering algorithm, allowing all possible values of *K* to be evaluated. The first step in the heuristic to determine the final value of *K* is to calculate the incremental *F* statistic (*incF*) [48] for each solution in *k*,

(4) $${\text{incF}}_{i}=\frac{\frac{R_{i}^{2}-R_{i-1}^{2}}{k_{i}-k_{i-1}}}{\frac{1-R_{i}^{2}}{n-(k_{i}-1)}}$$

where

(5) $$R^{2}=1-\text{(RSS/TSS)}.$$

*RSS* and *TSS* are the residual sum of squared error and total sum of squared errors, respectively, calculated for each cluster solution in *k* (*J*(*C*) from Eq. 3 is equal to *RSS*). *R*^2^ is an overall measure of the “fit” of the cluster solution to the original data. The incremental F statistic measures only the amount of fit gained from allowing an additional cluster in the solution, while also penalizing for adding this additional cluster. Because increasing *K* will almost certainly improve the *R*^2^ of the cluster solution, incF offers a measure that incorporates both fit and *K*. Initial candidate solutions are selected by identifying those with local maxima in *incF* (all solutions where *incF*_*k*_ > *incF*_*k*−1_ and *incF*_*k*_ > *incF*_*k*+1_).

After the initial candidate solutions are identified, a set of decision rules is employed to select the final value of *K*. Again, we stress that no method or technique provides a perfectly objective answer for the “correct” or “best” value of *K*. During the development of the methodology, an emphasis was placed on identifying *quantifiable* characteristics of the overall cluster configuration and the clusters themselves that could be used as a guide for this process. The HBSAC working group offered two qualifications for a suitable Hospital Group solution, 1) that no individual Hospital Group contains more than 20 hospitals and 2) that the number of “single hospital” Hospital Groups is minimized.

The decision rules are implemented via a three-step heuristic. First, all initial candidate solutions where any single Hospital Group contains more than 20 hospitals are removed. Next, for each of the remaining solutions, the number of single hospital Hospital Groups they contain is noted. The solution(s) having the minimum number of single hospital Hospital Groups is/are retained. If multiple solutions are retained from the previous step, the final step is to choose the candidate having the maximum *K* from the remaining solutions.

It is important to note that the decision rules presented here were created for use in a very specific application of the clustering methodology: to determine the number of Hospital Groups in Michigan. They are presented to illustrate a solution for determining *K* that integrates a statistical approach with a set of user-defined decision rules. The lack of a purely statistical metric to evaluate the correct number of clusters necessarily leads to subjectivity in the choice of *K*. However, by incorporating the two approaches, we have attempted to offer a semi-objective process to determine the number of clusters. With that being stated, any application of this clustering methodology where different types of observations or attribute data are employed needs to fully consider the use of the output clusters. Hence, the decision rules and the heuristic can and should be modified to suit the needs of the user.

### New hospital assignment

The new clustering methodology also provides a procedure to assign a new or prospective hospital to the existing set of Hospital Groups. In the Thomas Methodology, this task was accomplished re-running the entire methodology with market survey data (projected *RI* values for the new hospital) added as a new observation. Given the uncertainty and likely errors present in projected market surveys, we designed a method wherein a new hospital is assigned to an existing Hospital Group using only geographic location.

The geocoded location of the new hospital is required to calculate the travel distance from the new hospital to each existing hospital. These distances are placed in a 1 × *n* vector, which is rescaled using the maximum distance between any two hospitals in Michigan (see Input data) and arranged such that the entries are in the same order as the entries in the original travel distance matrix.

Like the Ward’s algorithm, the 2-step K-means + Ward’s algorithm produces a *K* × *m* matrix of cluster centers. The cluster centers from the Hospital Group solution are subset to only those columns corresponding to the travel distance attributes (column numbers *z*+1 to *m*), resulting in a *K* × *n* matrix. This subset represents the geographic location of the existing Hospital Group centers in *n*-dimensional space.

The Euclidean distance (*d*) from the new hospital to an existing Hospital Group center is calculated

(6) $$d=\sqrt{\overset{n}{\underset{i=1}{\sum }}{\left(c_{i}-h_{i}\right)}^{2}}$$

where *c*_*i*_ is the cluster center for the Hospital Group and *h*_*i*_ is the rescaled distance vector for the new hospital. A d value is calculated from the new location to each existing Hospital Group. The new hospital is assigned to the Hospital Group having the minimum *d* value.

## Results

We implemented the new Hospital Groups clustering methodology using inpatient hospitalization data from 2008 to 2010, which included 169 acute care hospitals. A small number of hospitals reported their inpatient data to the MIDB in tandem with another hospital or set of hospitals. The hospitals reporting together are owned by the same health care system and are located very near each other. Therefore, these were treated as a single observation for the purposes of clustering^d^. Two hospitals did not report any patient records to the MIDB and were removed prior to clustering. The final data matrix consisted of 158 observations with 1066 attributes (*CI* values for 906 zip codes and rescaled travel distance to 160 hospital locations).

**Figure 7:**
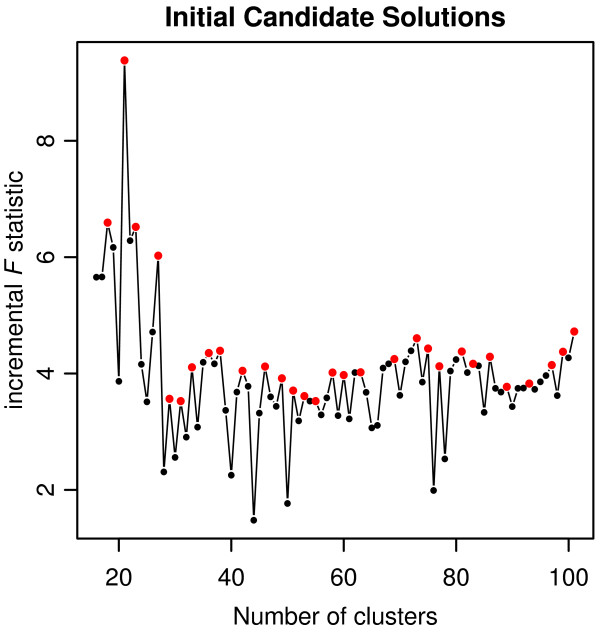
**Initial candidate solutions for Hospital Groups.** Data are truncated for display purposes. Red points represent local maxima in *incF* values.

**Figure 8:**
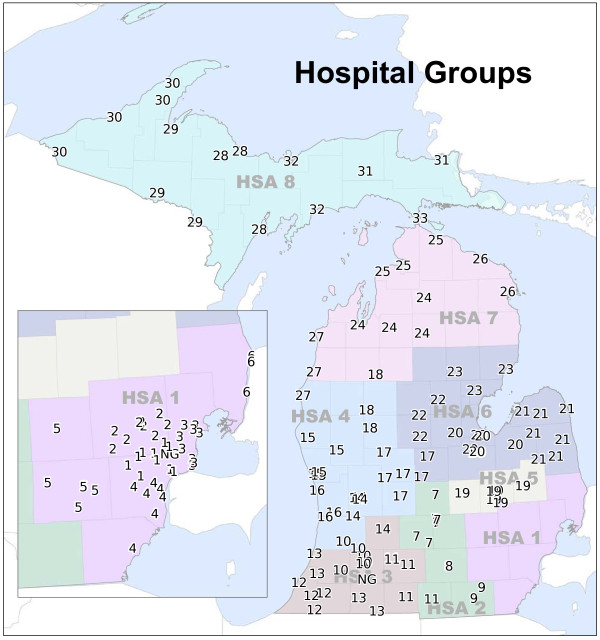
Hospital Groups created using new clustering methodology.

A Hospital Group solution was created using the 2-step K-means + Ward’s algorithm for each value of *K* from 2 to 157. We implemented the heuristic to select the number of Hospital Groups for the final solution. 52 initial candidate solutions were identified using *incF* values (see Figure 7 and Table 1). Next, candidate solutions having less than 29 clusters were removed due to the maximum number of hospitals in a single Hospital Group. From the remaining candidate solutions, the minimum number of single hospital Hospital Groups was 1. Therefore, all solutions greater than 33 clusters were removed from consideration. From the remaining candidate solutions, 33 was the maximum value of *K* and selected as the final Hospital Group solution (see Figure 8).

**Table 1:** Initial candidate solutions

Clusters	***incF***	**SH**	**Max**
3	94.81	0	91
5	81.11	0	60
7	33.59	0	61
11	14.88	0	51
14	7.93	0	48
18	6.59	0	45
21	9.38	0	36
23	6.52	0	36
27	6.02	1	24
29	3.56	1	17
31	3.52	1	17
33	4.11	1	17
36	4.35	2	17
38	4.39	5	17
42	4.05	7	17
46	4.12	9	16
49	3.92	11	16
51	3.71	13	12
53	3.61	15	12
55	3.53	16	12
58	4.02	20	12
60	3.97	22	12
63	4.02	27	12
69	4.25	36	12
73	4.61	40	12
75	4.43	43	12
77	4.13	44	9
81	4.38	48	8
83	4.17	49	7
86	4.29	53	7
89	3.77	58	7
93	3.83	64	7
97	4.14	70	7
99	4.37	73	7
101	4.72	77	7
103	5.22	79	7
107	4.94	83	7
109	3.77	86	7
112	3.79	90	7
115	3.84	93	7
120	3.41	99	7
124	3.08	103	5
126	2.52	107	5
129	2.44	111	5
132	2.39	114	5
135	2.10	118	5
141	1.91	125	5
144	1.91	129	4
146	1.87	133	4
149	1.99	136	3
152	1.88	141	3
154	1.92	145	3

SH are the number of single hospital clusters in the overall solution and Max is the maximum number of hospitals in any cluster in the Hospital Group solution. Solutions with less than 29 clusters have Max > 20 and were removed from consideration. From the remaining solutions, the minimum SH value was 1. Therefore, solutions with SH > 1 were removed from consideration. From the remaining 3 solutions (29, 31, 33), the 33 cluster solution was the maximum *K* and selected as the final Hospital Group solution.

To explore the stability of the Hospital Groups provided by the methodology, we re-created a 33 Hospital Group solution using data from 2005 to 2007. This allowed us to test the resulting Hospital Groups with data from an independent time period with no overlapping years. Because a small number of hospitals closed and opened during this time frame, after clustering, the hospitals were normalized such that only hospitals open during both time periods were compared. The normalization step was completed post-clustering as to not influence the results of the 2005-2007 Hospital Group solution. Overall, the two 33 Hospital Group solutions were in agreement on 89.76% of hospitals (149 of 166 hospitals). 24 of the 33 Hospital Groups produced using the 2005-2007 data were an *exact* match (in both group size and hospital membership) with their counterparts from the 2008-2010 data.

### Comparison to previous configuration

The overall fit of the original 64 Subarea configuration is slightly better than that of the 33 Hospital Group solution (*R*^2^ = 0.984 vs. *R*^2^ = 0.971). However, this is not entirely surprising, given that model fit is highly influenced the number of clusters. Further, *R*^2^ is influenced by the number of clusters with a single observation. The 64 Subarea configuration contains 32 of these clusters, while the 33 Hospital Groups only contains one such cluster; essentially, a single hospital cluster provides a perfect “fit”.

The *F* statistic is more appropriate for comparison as it incorporates the model fit (*R*^2^), while also penalizing for a larger number of clusters. We found that the 33 Hospital Group solution (*F* = 131.75) largely outperformed the original configuration (*F* = 92.86), which suggests that the new clustering methodology provides a more *efficient* set of Hospital Groups.

It is important to note that a direct comparison of the 64 Subarea and the 33 Hospital Group solutions can be somewhat misleading given that they were created with very disparate methods *and* do not have a similar number of clusters. Methods and procedures to evaluate clustering methods or algorithms generally compare cluster solutions with the same number of clusters or compare the cluster to solutions to a random clustering of observations.

## Discussion

Comparing the statistical fit of the 64 Subareas to the 33 Hospital Groups is not a fully appropriate approach to evaluating the new clustering methodology against the Thomas Methodology. In light of their final purpose for creating allocation units for the Michigan’s Bed Need Methodology, a more suitable evaluation is the performance of the methodologies themselves. In this, the small number of clusters produced by the Thomas Methodology, when implemented with up-to-date hospitalization data, speaks more to the overall utility of the methodology itself. Further, the application-oriented and conceptual concerns identified in the Thomas Methodology suggest that the principles of the methodology are outdated. Thus, the most basic advantage provided by the new clustering methodology is that it is theoretically valid, while also providing a usable Hospital Group solution.

Following an extensive review, the HBSAC and the state’s CON Commission recommended that the new clustering methodology for Hospital Groups be adopted into Michigan’s Hospital Bed Standards. Implementation of the new clustering methodology reduced the number of Hospital Groups in Michigan from 64 to 33. During development of the methodology, an emphasis was placed on reducing the number of single hospital Hospital Groups, thereby assuming a more regional view of community-based need than the previous configuration. In the 33 Hospital Group solution, only one Hospital Group contains a single hospital (2.86% of the groups). This result was substantially different than the former configuration wherein 50% of the 64 Subareas contained a single hospital. While the initial move toward more regional-level planning and regulation units is consistent with other states’ CON programs, the actual consequences for inpatient hospital bed distribution and access in Michigan remain to be seen. We are encouraged, however, by preliminary tests showing that the 33 Hospital Group configuration did not substantially alter predictions of the state’s future bed demand.

An issue to consider is the use of alternative or additional data for clustering hospitals in Michigan. Because the focus of this application is to define Hospital Groups for inpatient hospital bed planning, we chose only to include inpatient hospitalization data. However, other measures such as the American Hospital Association’s case-mix adjusted discharges may be explored in the future. Adjusted discharges incorporate both inpatient and outpatient hospital visits, possibly offering a more complete characterization of community health care utilization. Additionally, raw inpatient days do not provide insight into the efficacy of the hospitalizations or their overall contribution to public health [49]. The use of inpatient hospitalization data limits the scope of the analysis. Future research exploring alternative data sources for clustering hospitals would likely prove beneficial.

Although the clustering methodology was designed for the specific purpose of creating groups of hospitals, the methodology relies upon theoretically sound and generally accepted approaches to cluster analysis. Therefore, it is highly transferable for other clustering applications. The methodology is suitable for use with a variety of data sources, places, or scales of analysis and, importantly, can be used to create small areas.

Georeferenced utilization data is the fundamental data source for the clustering methodology. Although we employed inpatient hospitalization data, the methodology does not require this specific data source. Any utilization data that can be georeferenced, separating utilization into source and destination locations, and can be converted to *CI* values is appropriate. These data may include, but are not limited to, outpatient visits, primary care visits, or Medicare data.

The clustering methodology is not scale dependent, nor dependent upon a specific place. Given georeferenced utilization data, the methodology will provide coherent results for any region or state. This also holds true for use in other countries, given the appropriate input data. Because the 2-step K-means + Ward’s algorithm does not require a substantial amounts of computer processing power, large datasets can be analyzed efficiently by the methodology. This provides an opportunity to expand the study domain to include multiple states or districts or to conduct a nation-wide analysis.

One of the most notable topics in health services research over the past 30 years has been the exploration of small area variation in health care utilization [21,50], spending [51], and outcomes [52] in the US. These studies often rely on an aggregation method wherein *small areas* are formed by grouping disaggregated population units into larger regions based on similarity in health services use. The method implemented by Wennberg and colleagues at Dartmouth employs a simple plurality rule, grouping areal units based on a single *CI* value, not their overall patterns of utilization [21]. In rural communities, this process is generally straightforward considering that much of the population’s health care needs are provided by a single facility. Because urban areas often contain a greater number of facilities, service use by any given community is often distributed similarly among facilities [30], complicating small area creation and/or service area definition. Using our clustering methodology, community utilization patterns can be expressed as the *CI* values from areal units to hospitals. The areal units could then be clustered into regions of similar hospital use, where the overall utilization patterns and location are considered. However, we note that an additional step would be required to link the clustered areas to specific hospitals or groups of hospitals using this methodology.

## Conclusions

The goal of our new clustering methodology to create Hospital Groups was for it to be ***objective***, ***replicable***, and ***sustainable***. Given the politically charged climate surrounding CON regulation in Michigan, a full recasting of the theoretical approach to cluster hospitals was no small undertaking. We believe that placing our focus on the concepts of hospital similarity and the theoretical underpinnings of the methods, rather than results, allowed for a politically objective overall methodology to emerge. In addition, we implement a heuristic that selects the final number of Hospital Groups based on desirable characteristics of the solutions instead of relying on a predefined number. The use of a heuristic does not completely remove all subjectivity from our methodology. However, by including the decision rules in the methodology, the new clustering methodology provides a level of transparency that was not present in the post-clustering modification step of the previous methodology.

Two distinct interpretations of “replicable” are fulfilled by the clustering methodology. First, by integrating the K-means and Ward’s clustering algorithms, we have effectively removed the unconstrained stochastic element associated with random starting locations in K-means. Each time the methodology is run with the same data, it will produce the same final Hospital Group solution (both the configuration of the Hospital Groups and the number of Hospital Groups). By supplying the methodology’s source code, we have provided an unambiguous representation. Further, the methodology is built upon well-known clustering algorithms allowing it be transferable to other statistical packages and applications.

We examined the sustainability of the clustering methodology by creating a 33 Hospital Group solution using hospitalization data from 2005-2007. The high level of agreement in the composition and size of the resulting Hospital Groups suggests that the methodology captures long-term community hospital utilization patterns in Michigan. Therefore, when the clustering methodology is run in the future, Hospital Group configuration will not change dramatically unless community utilization patterns have significantly changed.

We believe that the appropriate levels of consideration were given to the scientific, practical, and political concerns encountered during the developmental process. The new clustering methodology offers substantial improvement over the previous methodology, as it is unambiguously actionable and produces superior results. Furthermore, the methodology is generalizable such that it is suitable for clustering both facilities or areal units, for use with a variety of data sources, and for use within a variety of health care service applications.

## Endnotes

^a^Interpreting the definition of the “home areal unit” of each hospital or cluster of hospitals in the Thomas Methodology was especially problematic. The original manuscript is quite vague in its discussion of home areal units. Unfortunately, the definition in the Hospital Bed Standards does not offer clarification. Therefore, we implemented multiple versions of the Thomas Methodology, each with a slightly different interpretation of the home areal unit. Although each produced unique results, none provided Subareas that were similar to the current configuration. The results presented in Figure 3 defined the home areal unit as the zip code in which the hospital is located. This implementation also allowed the algorithm to run until clustering was completed.

^b^Although not discussed in the manuscript, the CON approved source code contains additional steps to assign a numeric identifier to the resulting Hospital Groups based on their geographic location and bed inventory.

^c^Based on *K*^*N*^/*K*! [53] where *K* = 50 and *N* = 158.

^d^Because these hospitals were each associated with a unique geographic location, their travel distance measurements were slightly dissimilar. To calculate the travel distances for the grouped set, we took the mean of the hospitals comprising the group. However, when calculating the number of “single hospital” Hospital Groups during the clustering methodology, the grouped set was not considered a single facility.

## Competing interests

The authors declare that they have no competing interests.

## Authors’ contributions

PLD and AMS assisted in designing the clustering methodology, writing the R code, and preparing the manuscript. JPM assisted in designing the clustering methodology and preparing the manuscript. All authors read and approved the final manuscript.

## Pre-publication history

The pre-publication history for this paper can be accessed here:

http://www.biomedcentral.com/1472-6963/13/333/prepub

## Supplementary Material

Additional file 1 **The Thomas Methodology.** R code to implement our interpretation of the methodology provided by Thomas et al. [30]. Click here for file

Additional file 2 **Michigan’s Hospital Group Methodology.** R code to implement the new clustering methodology. Includes the 2-step K-means + Ward’s clustering algorithm, along with the heuristic for determining the number of Hospital Groups and the steps required to assign a new hospital to the existing Hospital Groups. Click here for file
