# Supplementary material for: Regional health care planning: a methodology to cluster facilities using community utilization patterns
Source: BMC Health Serv Res. 2013 Aug 22;13:333. doi: 10.1186/1472-6963-13-333 (PMC3766152; doi:10.1186/1472-6963-13-333)
Supplement: Additional file 1 — The Thomas Methodology. R code to implement our interpretation of the methodology provided by Thomas et al. [30]. [file 1472-6963-13-333-S1.pdf]

```
########################################################################
####                                                               ####
####  Additional file 1                                            ####
####                                                               ####
####  R code to implement the Thomas Methodology                   ####
####                                                               ####
####  Citation information (code)                                  ####
####  Delamater PL, Shortridge AM, and JP Messina, Regional health ####
####  care planning: a methodology to cluster facilities using     ####
####  community utilization patterns. BMC Health Services Research ####
####                                                               ####
####  Citation information (original methodology)                  ####
####  Thomas JW, Griffith JR, and P Durance, Defining hospital     ####
####  clusters and associated service communities in metropolitan ####
####  areas, Socio-Economic Planning Sciences 1981, 15(2):45-51    ####
####                                                               ####
####  Max Relevance Algorithm Clustering Algorithm                 ####
####                                                               ####
####  Requires: Patient visits table (zip -> hospital)             ####
####            Zip population data                                ####
####            Hospital info table                                ####
####            Hospital zip code table                            ####
####                                                               ####
####  Interpreted and converted to R code by Paul Delamater and    ####
####  Ashton Shortridge during summer, 2011 for the Michigan       ####
####  Hospital Bed Standard Advisory Committee working group.      ####
####  Funding for this research was provided by the Michigan       ####
####  Department of Community Health.                              ####
####                                                               ####
########################################################################


########################################################################
##                                                                    ##
##  Get input data                                                    ##
##                                                                    ##
########################################################################


###########################
## Read patient visits data
###########################

####  Note: pv is a table with hospitals in rows and zip codes
####        in columns. Hospital identifier column should be
####        labeled "HOSP_ID". Zip code column lables should
####        be the five digit zip code (e.g., "48823"). Table
####        entries are the number of hospitalizations from
####        residents of each zip code at each hospital.

# Load data
pv <- read.csv("inputdata/hosp.zip.visits.mtx.csv")

# Ensure HOSP_ID in character format
pv$HOSP_ID <- as.character(pv$HOSP_ID)

# Remove characters from column names
# R adds an "X" to the zip code number
# Assumes all zip codes are 5 digits
names(pv)[2:ncol(pv)] <- substr(as.character(names(pv)[2:ncol(pv)]), 2, 6)

############################################################
```

```
## Convert number of visits to proportions (Relevance Index)
###########################################################

# Define variable for last zip code column
n.zip <- ncol(pv)

# Sum hospital visits for each zip code
# Assumes HOSP_ID is first column
zip.visits <- colSums(pv[,2:n.zip])

# Divide each entry by summed visits to create Rij values
pv[,2:n.zip] <- pv[,2:n.zip] / rep(zip.visits, each = nrow(pv))

################################
## Read hospital attributes data
################################

####  Note: hosp.info is a table with hospitals in rows and
####        attributes in columns. In this case, the column
####        that corresponds to the patient records is "MIDB".

# Load data
hosp.info <- read.csv("inputdata/hospitals.csv")

####  Note: hosp.HAU is a table with hospitals in rows and
####        attributes in columns. In this case, the column
####        that corresponds to the patient records is "MIDB".
####        Home Areal Unit is "ZIP".

# Load home areal unit (zip code) of each hospital
hosp.HAU <- read.csv("inputdata/hospital.zipcodes.csv")

# Attach to home areal unit to patient records
pv <- merge(pv, hosp.HAU, by.x="HOSP_ID", by.y="MIDB", all.x=TRUE)

# Change column name
names(pv)[ncol(pv)] <- "HAU"

# Add column with Rij (Relevance Index) of each hospital in
# its own home areal unit
for (h in 1:nrow(pv)) pv$RiHAU[h] <- pv[h,which(names(pv)==pv$HAU[h])]

###############################
## Get zip code population data
###############################

####  Note: zip pop is a table with the zip code name in a
####        column, "ZIP" and the population of the zip code
####        in a column, "POP"

# Load data
zip.pop <- read.csv("inputdata/zipcode.population.csv")


########################################################################
##                                                                    ##
## Code to implement Thomas Methodology                               ##
##                                                                    ##
########################################################################


#######################################
```

```
## Prepare data and create data holders
########################################

# Add column with initial alpha values (all are set at 0.02)
# In an update of this code, initial alpha values are set at 0.05
pv$alpha <- 0.02

# Define initial values for alpha variables
alpha.1 <- 0.02
alpha.2 <- 0.125

# Add binary holder column for individuals / groups
pv$Group <- 0
# Add column to hold hospital names after clustering
pv$GrNames <- pv$HOSP_ID

#####################################################
## Calculate population weighted relevence index, Rj
#####################################################

####  Note: Pi = population of areal unit i
####        Rij = relevance index values for areal unit i
####        to hospital j

# Calculate PiRij values (Pi * Rij)
PiRij.matrix <- pv
PiRij.matrix[,2:n.zip] <- PiRij.matrix[,2:n.zip] * rep(zip.pop$POP, each =
      nrow(PiRij.matrix))

# Create holder for Rj values
Rj.all <- NULL

# Create holder for Ij zip codes
Ij.matrix <- pv
Ij.matrix[,2:n.zip] <- 0

# Calculate Rj for each hospital
for (j in 1:nrow(pv)) {

  # Get hospital j's Ri values
  hosp.j <- pv[j,2:n.zip]

  ####  Note: From Thomas et al., Ij = set of areal units
  ####        for which individual relevance values of
  ####        hospital j exceeds or equals alpha

  # Find zip codes with Rij greater than alpha
  Ij.list <- which(hosp.j >= alpha.1)+1

  # Write zip codes greater than alpha to Ij holder
  Ij.matrix[j,c(Ij.list)] <- 1

  # If no areal units in Ij, Rj value is zero
  if (length(Ij.list) == 0) {

    # Write hospital ID and 0 to Rj holder
    Rj.all <- rbind(Rj.all, cbind(as.character(pv$HOSP_ID[j]), 0))

    } else {


  # Get list of zip code names
```

```
  Ij.zips <- names(pv)[Ij.list]

  # Get numerator value for Rj
  PiRij <- sum(PiRij.matrix[j,Ij.list])

  # Get denominator value for Rj (total zip code population)
  Pi <- sum(zip.pop$POP[c(Ij.list-1)])

  ####  Note: Rj = sum(Pi(dij/Di)) / sum(Pi)
  ####        where dij/Di is Relevance Index

  # Calculate Rj (population weighted relevance index)
  Rj <- PiRij / Pi

  # Put in holder
  Rj.all <- rbind(Rj.all, cbind(as.character(pv$HOSP_ID[j]), Rj))

  }

}

# Make Rj.all into dataframe
Rj.all <- as.data.frame(Rj.all)

# Rename columns in Rj.all
names(Rj.all) <- c("HOSP_ID", "Rj")

# Convert from factor to numeric and character
Rj.all$Rj <- as.numeric(levels(Rj.all$Rj)[Rj.all$Rj])
Rj.all$HOSP_ID <- as.character(Rj.all$HOSP_ID)

###################################################
## Remove hospitals with Rj of 0 from analysis
## These hospitals are ungroupable using the method
###################################################

# Locate hospitals with Rj = 0
zeros <- which(Rj.all$Rj == 0)

# Get hospital ID
Ungroupable.hospitals <- Rj.all[c(zeros),1]

# Remove hospitals from matrices
pv <- pv[-c(zeros),]
Rj.all <- Rj.all[-c(zeros),]
Ij.matrix <- Ij.matrix[-c(zeros),]
PiRij.matrix <- PiRij.matrix[-c(zeros),]

# Write ungroupable hospitals info to table
Ungroupable.hospitals.info <- hosp.info[hosp.info$MIDB %in% Ungroupable.hospitals, ]

###########################################################
##  Start iterative process part of the code and explicitly
##  state which method will be used to STOP the process
###########################################################

# Create holder for grouped hospitals
Grouped.Hospitals <- NULL

# Create holder for temporary Rj.min values
Rj.temp <- NULL
```

```
####  Note: From Thomas et al., The procedure terminates
####        when one of three conditions occurs: (1) all
####        hospitals have been aggregated into a single
####        large cluster; (2) a user-specified number of
####        iterations has been completed; or (3) all
####        identified clusters are stable, i.e., no
####        cluster serves more than alpha of the patients
####        in the home areal unit of any other cluster.
####
####        These lines will make the iterative process stop
####        at a specified number of Subareas, similar to
####        option number (2) above. To choose this option
####        uncomment the following lines and comment out,
####        "run <- 1" and "while (run == 1) {"

# Select desired number of Subareas
# n.subareas <- 64
# Start grouping hospitals
# while (nrow(pv) > n.subareas) {

####        These lines will make the iterative process stop
####        when no hospital/group has greater than alpha
####        of any other hospitals/group's home area (option
####        number (3) above). These line also stops code if
####        all hospitals are aggregated into one large
####        group (option number (1) above).

# Create variable used in the iterative process for stopping
run <- 1

# Start grouping hospitals
while (run == 1) {

  ################################
  ## Find hospital with minimum Rj
  ################################

  ####  Note: Checks for hospitals in a temporary holder. This
  ####        holder is defined below. It is used in case any
  ####        hospital is the min Rj, but does not have another
  ####        hospital to group with yet

  if (length(Rj.temp) == 0) {

    # Locate hospital with minimum Rj value
    which.hosps <- which(Rj.all$Rj == min(Rj.all$Rj))

    # Get number of "minimum" Rj hosps
    n.min.hosps <- length(which.hosps)

    } else {

      # Locate hospital with minimum Rj value (minus temp)
      which.hosps <- which(Rj.all$Rj == min(Rj.all$Rj[-Rj.temp]))

      # Get number of "minimum" Rj hosps to determine if ties exist
      n.min.hosps <- length(which.hosps)

    }


  # If a tie exists, randomly select which of the hospitals is
```

```
# is selected for aggregation.  Otherwise min.hosp is used
if (n.min.hosps > 1) {

  # Create random variable using number of tied hospitals
  min.hosp <- round((n.min.hosps-1)*runif(1))+1

  # Select hosptial using random variable
  min.hosp <- which.hosps[min.hosp]

  } else {

    # Use the single hospital
    min.hosp <- which.hosps

  }

# Subset minimum hospital from Rj.all
Rj.min <- Rj.all[min.hosp,]

# Print to screen to display which hospital is selected
print(paste("Rj.min = ", Rj.min[2], ", HOSP_ID = ", Rj.min[1], sep=""))

# Get Rj.min's home areal unit (column number!)
Rj.min.Ij <- which(names(pv) == pv$HAU[min.hosp])

# Print Rj.min's home areal unit and RI
print(paste("Rj.min HAU = ", pv$HAU[min.hosp], ", RI = ", pv[min.hosp, Rj.min.Ij],
    sep=""))

################################################################
## Find hospital/cluster with max RI in Rj min's home areal unit
################################################################

####  Note: From Thomas et al., the hospital with the smallest
####        Rj is identified and grouped to form a cluster with
####        the hospital having the greatest individual
####        relevance in hospital j's home areal unit.

# Find max RI in Rj min's home areal unit
Rj.max.Rj.min <- which(pv[,Rj.min.Ij] == max(pv[,Rj.min.Ij]))

# If statement in case it selects itself
# e.g., no hospital or cluster has higher Ri in minimum's
# home area
if (Rj.max.Rj.min == min.hosp) {

  # Pick the next highest after removing min hospital
  next.Rj.max <- max(pv[-min.hosp,Rj.min.Ij])
  Rj.max.Rj.min <- which(pv[,Rj.min.Ij] == next.Rj.max)

  }

# In case of ties for Rj.max select randomly from tied hospitals
if (length(Rj.max.Rj.min) > 1) {

  # Generate random number
  rand <- round((length(Rj.max.Rj.min)-1)*runif(1))+1

  # Use random number to select
  Rj.max.Rj.min <- Rj.max.Rj.min[rand]

  }
```

```
# Get RI of Rj.max
alpha.Rj.max <- pv[Rj.max.Rj.min, Rj.min.Ij]

# Print alpha value and HOSP ID to screen
print(paste("alpha.Rj.max = ", alpha.Rj.max, ", HOSP_ID = ", pv$HOSP_ID[Rj.max.Rj.min],
    sep=""))

################################################################
## Big logic part of code. Determines whether to group hospitals
## or move to next minimum hospital in list
################################################################

####  Note: From Thomas et al., ...the hospital with the smallest
####        Rj is identified and grouped to form a cluster with
####        the hospital having the greatest individual relevance
####        in hospital j's home areal unit.
####
####        We assume that there is a 'cut-off' value in this
####        step based on the text in termination option number
####        (3), i.e., no cluster serves more than alpha of the
####        patients in the home areal unit of any other cluster.

# If the Rj value in Rj.min's home area is larger than the
# alpha cutoff of the hospital or cluster, then cluster
if (alpha.Rj.max >= pv$alpha[Rj.max.Rj.min]) {

  #########################################
  ## Update RI values to reflect clustering
  #########################################

  # Sum RI values for clustered hospitals
  pv[Rj.max.Rj.min,2:n.zip] <- pv[Rj.max.Rj.min,2:n.zip] + pv[min.hosp,2:n.zip]

  # Update alpha score and group columns
  pv$alpha[Rj.max.Rj.min] <- alpha.2
  pv$Group[Rj.max.Rj.min] <- 1
  pv$GrNames[Rj.max.Rj.min] <- paste(pv$GrNames[Rj.max.Rj.min], pv$GrNames[min.hosp],
    sep=",")

  #####################################
  ## Update home areal unit for cluster
  #####################################

  ####  Note: From Thomas et al., When a previously formed cluster
  ####        j* is identified for further clustering, its home
  ####       areal unit is assumed to be the home areal unit of
  ####        the hospital (member of j*) having the highest Rij
  ####        among the cluster hospitals' home areas

  # If Rj min's relevance in its home area is larger than Rj max
  # assign new home areal unit to newly formed cluster
  if (pv$RiHAU[Rj.max.Rj.min] < pv$RiHAU[min.hosp]) {

    # Assign Rij to cluster entry
    pv$RiHAU[Rj.max.Rj.min] <- pv$RiHAU[min.hosp]

    # Assign new home areal unit to cluster entry
    pv$HAU[Rj.max.Rj.min] <- pv$HAU[min.hosp]

  }
```

```
#########################################################
## Update Ij.matrix to reflect new alpha value of cluster
#########################################################

# Find zip codes above new alpha value
Ij.new <- which(pv[Rj.max.Rj.min,2:n.zip] >= alpha.2)+1

# Clear old Ij row, then write new zip codes to Ij holder
Ij.matrix[Rj.max.Rj.min, 2:n.zip] <- 0
Ij.matrix[Rj.max.Rj.min,c(Ij.new)] <- 1

######################
## Update PiRij.matrix
######################

# Sum PiRij entries
PiRij.matrix[Rj.max.Rj.min,2:n.zip] <- PiRij.matrix[Rj.max.Rj.min,2:n.zip] +
  PiRij.matrix[min.hosp,2:n.zip]

##############################################
## Update Rj.all with new list of Ij zip codes
##############################################

# Get numerator value for Rj
n.PiRij <- sum(PiRij.matrix[Rj.max.Rj.min,Ij.new])

# Get denominator value (total zip code population)
n.Pi <- sum(zip.pop$POP[c(Ij.new-1)])

# Calculate Rj  (population weighted relevence index)
Rj <- n.PiRij / n.Pi

# Put in holder
Rj.all$Rj[Rj.max.Rj.min] <- Rj

#########################################################
## Remove Rj.min from pv, Ij.matrix, PiRij.matrix, Rj.all
## because it has now been grouped
#########################################################
pv <- pv[-c(min.hosp),]
Ij.matrix <- Ij.matrix[-c(min.hosp),]
PiRij.matrix <- PiRij.matrix[-c(min.hosp),]
Rj.all <- Rj.all[-c(min.hosp),]

# Write Rj.min hosp to holder
Grouped.Hospitals <- c(Grouped.Hospitals, Rj.min$HOSP_ID)

# Print to screen which hospitals have been grouped
print(Grouped.Hospitals)

# Reset Rj.temp because current Rj.min has been grouped
Rj.temp <- NULL

} else {

  ###############################################################
  ## Re-run steps with a different Rj.min because aggregation may
  ## produce clusters with home areas > alpha) in former Rj min's
  ## home area.  So we hold onto this Rj.min and re-check later
  ## List this Rj.min in holder
  ###############################################################
```

```
      Rj.temp <- c(Rj.temp, min.hosp)

    }

  # Print to screen which hospitals are in Rj.temp and
  # the length of both Rj.temp and Rj.all
  print(paste("Rj temp has: ", length(Rj.temp), " hospitals/cluster", sep=""))
  print(paste("Rj all has: ", nrow(Rj.all)-1, " hospitals/clusters remaining", sep = ""))

  ##################################################
  ## Determine whether to keep attempting to cluster
  ## or to terminate the iterative process
  ##################################################

  # If all the hospitals (-1) are in Rj.temp, then no
  # hospital has more than alpha of another's home area
  if (length(Rj.temp) == nrow(Rj.all)-1) {
    run <- 0
  }

  # If all hospitals are grouped Rj.all has one row
  if (nrow(Rj.all) == 1) {
    run <- 0
  }

}

##################################################
## Attach Subarea designation to hospital info file
##################################################

# Get number of Subareas
n.subareas <- dim(pv)[1]

# Make empty holder
subarea.table <- NULL

# Break apart output table from Thomas method
# and insert into holder
for (p in 1:n.subareas) {
  names <- unlist(strsplit(pv$GrNames[p], ","))
  subarea.table <- rbind(subarea.table, cbind(p, names))
}

# Rename column names
colnames(group.table) <- c("Thomas", "MIDB")

# Attach Subarea names to hospital info file
hosp.info <- merge(hosp.info, group.table, by="MIDB", all.x=TRUE)

# Name ungroupable hospitals "NG"
hosp.info$Thomas <-as.character(hosp.info$Thomas)
hosp.info$Thomas[is.na(hosp.info$Thomas)] <- "NG"
```
