# Supplementary material for: Regional health care planning: a methodology to cluster facilities using community utilization patterns
Source: BMC Health Serv Res. 2013 Aug 22;13:333. doi: 10.1186/1472-6963-13-333 (PMC3766152; doi:10.1186/1472-6963-13-333)
Supplement: Additional file 2 — Michigan’s Hospital Group Methodology. R code to implement the new clustering methodology. Includes the 2-step K-means + Ward’s clustering algorithm, along with the heuristic for determining the number of Hospital Groups and the steps required to assign a new hospital to the existing Hospital Groups. [file 1472-6963-13-333-S2.pdf]

```
########################################################################
####                                                               ####
####  Additional file 2                                            ####
####                                                               ####
####  R code to implement the new clustering methodology           ####
####                                                               ####
####  Citation information                                         ####
####  Delamater PL, Shortridge AM, and JP Messina, Regional health   ####
####  care planning: a methodology to cluster facilities using      ####
####  community utilization patterns. BMC Health Services Research   ####
####                                                               ####
####  2-step K-means + Ward's Algorithm                            ####
####                                                               ####
####  Requires: Patient visits table (zip -> hospital)             ####
####            Hospital travel distance table (hosp -> hosp)      ####
####            Hospital info table                                ####
####                                                               ####
####  Methodology developed by Paul Delamater, Ashton Shortridge,   ####
####  and Joe Messina during summer, 2011 for the Michigan Hospital  ####
####  Bed Standard Advisory Committee working group. Funding for   ####
####  this research was provided by the Michigan Department of     ####
####  Community Health.                                            ####
####                                                               ####
########################################################################


########################################################################
##                                                                    ##
##  Get input data                                                    ##
##                                                                    ##
########################################################################


##########################
## Read patient visits data
##########################

#### Note:  pd.1, pd.2, pd.3 are tables with hospitals in rows
####        and zip codes in columns. Hospital identifier column
####        should be labeled "HOSP_ID". Zip code column lables
####        should be the five digit zip code (e.g., "48823").
####        Table entries are the number of patient days from
####        residents of each zip code at each hospital.
####
####        Assumes patient day matrices have similar dimensions!

# Load data
pd.1 <- read.csv("inputdata/hosp.zip.patdays.mtx.y1.csv")
pd.2 <- read.csv("inputdata/hosp.zip.patdays.mtx.y2.csv")
pd.3 <- read.csv("inputdata/hosp.zip.patdays.mtx.y3.csv")

# Create 3 year sum matrix
p.sum.3yr <- pd.1[,2:ncol(pd.1)] + pd.2[,2:ncol(pd.2)] + pd.3[,2:ncol(pd.3)]

# Re-attach hospital names column
p.sum.3yr <- cbind(pd.1[,1], p.sum.3yr)

# Rename hospital names column
names(p.sum.3yr)[1] <- "HOSP_ID"


# Ensure HOSP_ID in character format
```

```
p.sum.3yr$HOSP_ID <- as.character(p.sum.3yr$HOSP_ID)

# Remove characters from column names
# R adds an "X" to the zip code number
# Assumes all zip codes are 5 digits
names(p.sum.3yr)[2:ncol(p.sum.3yr)] <- substr(as.character(names(p.sum.3yr)
      [2:ncol(p.sum.3yr)]), 2, 6)

###############################################################
## Convert raw patient days to proportions (Commitment Index)
###############################################################

# Define variable for last zip code column
n.zip <- ncol(p.sum.3yr)

# Sum patient days for each hospital
# Assumes HOSP_ID is first column
hosp.pat <- rowSums(p.sum.3yr[,2:n.zip])

# Divide each column by total patient days
p.sum.3yr[,2:n.zip] <- p.sum.3yr[,2:n.zip] / hosp.pat

# Rename table
p.CI.3yr <- p.sum.3yr
rm(p.sum.3yr)

###########################################
## Remove hospitals with no patient visits
###########################################

# Locate hospitals with zero visits
zero.pv <- which(hosp.pat == 0)

# Get names of zero hospitals (will need this later!)
zero.names <- as.character(p.CI.3yr$HOSP_ID[zero.pv])

#### Note:  p.CI.3yr is now an n x z+1 matrix of CI values.
####        The "+1" includes the identifier column (HOSP_ID).

# Remove hospitals from CI matrix
p.CI.3yr <- p.CI.3yr[-c(zero.pv),]

#################################
## Read hospital attributes data
#################################

#### Note:  hosp.info is a table with hospitals in rows and
####        attributes in columns. In this case, the column
####        that corresponds to the patient records is "MIDB".

# Load data
hosp.info <- read.csv("inputdata/hospitals.csv")

############################
## Read travel distance data
############################

#### Note:  od is a table with "TO", "FROM", and "DISTANCE"
####        as columns (format from ArcGIS Network Analyst).
####        This table must be re-arranged such that it is
####        an actual OD matrix (n x n dimensions). If data
####        is already arranged in an OD matrix, skip to
```

```
####        "Scale table" section.

# Load data
od <- read.csv("inputdata/travel-distance.csv")

#############################
## Convert table to OD matrix
#############################

# Create empty holder
dist.mat <- NULL

# Get unique FROM hospitals
f.hosp <- unique(od$FROM)

# Loop through hospitals
for (fr in 1:length(f.hosp)) {
  # Subset
  od.sm <- od[od$FROM == f.hosp[fr],]
  # Sort matrix, shouldn't be necessary... but safer
  od.sm <- od.sm[order(od.sm$TO),]
  # Append distance to holder as a ROW
  dist.mat <- rbind(dist.mat, od.sm$DISTANCE)
}

# Make into dataframe
dist.mat <- as.data.frame(dist.mat)

# Assign column names
names(dist.mat) <- f.hosp

# Assign row names
row.names(dist.mat) <- f.hosp

###########################################
## Scale table to match CI data range (0-1)
###########################################

# Get maximum distance between hospitals
max <- max(dist.mat)

# Rescale data
dist.mat <- dist.mat/max

##############################################
## Join distance data matrix to CI data matrix
##############################################

#### Note:  To create the final n x m data matrix used for
####        clustering, the n x z and n x n matrix are joined.

# Add column for table join
dist.mat$HOSP_ID <- row.names(dist.mat)

## Join tables, add distance matrix to CI data
p.CI.3yr <- merge(p.CI.3yr, dist.mat, by="HOSP_ID")



#########################################################################
```

```
##                                                                        ##
##  Custom 2-step K-means + Ward's clustering function                   ##
##                                                                        ##
##########################################################################

#### Note:  The inputs for the function are the n x m data
####        matrix (x) and the desired number of clusters (clusters).

kmeans.ward <- function(x, clusters) {

  # Create distance matrix
  d <- dist(x, "euclidean")

  # Perform Ward's clustering
  hc <- hclust(d, method="ward")

  # Get cluster members at "K" clusters
  memb <- cutree(hc, k = clusters)

  # Make empty holder for cluster center locations
  cent <- NULL

  # Get cluster centers
  for (k in 1:clusters) {
    cent <- rbind(cent, colMeans(x[memb == k,]))
  }

  # Use cluster centers from Ward's to seed K-means clustering
  k.m <- kmeans(x, cent, iter.max = 10000)

  # Return the K-means object
  return(k.m)

}


##########################################################################
##                                                                        ##
##  Create initial cluster solutions for Hospital Groups                 ##
##                                                                        ##
##########################################################################

#######################################
## Prepare data and create data holders
#######################################

#### Note:  All possible numbers of clusters are considered
####        from 2 to n-1.

# Define the range of cluster solutions to evaluate (the set k)
cl.max <- nrow(p.CI.3yr)-1
clusters <- c(2:cl.max)

# Create an empty holder for cluster statistics
wss <- bss <- r2 <- incF <- SingHosp <- MaxSize <- rep(0, length(clusters))
k.data.pat <- cbind(clusters, wss, bss, r2, incF, SingHosp, MaxSize)

# Get number of data attributes in table (columns)
col.max <- ncol(p.CI.3yr)


#####################################################
```

```
## Conduct K-means + Ward's for all cluster solutions
#####################################################

for (K in 1:length(clusters)) {

  # Use K-means + Wards method to create clusters
  Kclust <- kmeans.ward(p.CI.3yr[,2:col.max], clusters[K])

  # Write cluster statistics to data holder
  # Within sum of squares
  k.data.pat[K,2] <- Kclust$tot.withinss

  # Between sum of squares
  k.data.pat[K,3] <- Kclust$betweenss

  # R^2
  k.data.pat[K,4] <- 1-(Kclust$tot.withinss/Kclust$totss)

  # Number of single hosp clusters
  table.c <- table(Kclust$cluster)
  k.data.pat[K,6] <- sum(table.c == 1)

  # Maximum size of any single cluster
  k.data.pat[K,7] <- max(table.c)

}

# Convert data holder to dataframe
k.data.pat <- as.data.frame(k.data.pat)

#################################
## Calculate incremental F scores
#################################

n.obs <- nrow(p.CI.3yr)

for (i in 2:length(clusters)) {

  k.data.pat$incF[i] <- ((k.data.pat$r2[i]-k.data.pat$r2[i-1])/(k.data.pat$clusters[i]-
      k.data.pat$clusters[i-1])) /  ((1-k.data.pat$r2[i])/((n.obs)-
      (k.data.pat$clusters[i]-1)))

}


########################################################################
##                                                                    ##
##  Select the number of Hospital Groups using the heuristic          ##
##                                                                    ##
########################################################################


###########################################
## Find local maxima in incremental F scores
###########################################

# Make variable of the last candidate solution to evaluate for maxima
i <- cl.max-1



# Find the local maxima
```

```
incF.peaks <- which(k.data.pat$incF[3:i] > k.data.pat$incF[2:(i-1)] &
      k.data.pat$incF[3:i] > k.data.pat$incF[4:(i+1)])+2

# Subset initial candidate solutions
candidates <- k.data.pat[incF.peaks,]

########################################################################
## Remove solutions wherein a single cluster has more than 20 hospitals
########################################################################

candidates <- candidates[candidates$MaxSize < 20,]

############################################################
## Subset solutions to those with the "minimum" number of
## single hospital Hospital Groups from remaining solutions
############################################################

candidates <- candidates[candidates$SingHosp == min(candidates$SingHosp), ]

#######################################
## From the remaining solutions select
## the solution with most clusters, K
#######################################

solution <- candidates[candidates$clusters == max(candidates$clusters), ]

# Get number of clusters
n.clusters <- solution$clusters

####################################################
## Use K-means + Wards method to re-create clusters
####################################################

#### Note:  Only the cluster statistics were kept in the
####        initial clustering process. The final cluster
####        solution is recreated to extract Hospital Group
####        membership and cluster center information.
####        Because the clustering algorithm provides
####        deterministic results, this clustering
####        configuration will be identical to the one
####        formed in the intial clustering process.

HG.solution <- kmeans.ward(p.CI.3yr[,2:col.max], n.clusters)

# Attach Hospital Group number to MIDB name
HG.names <- as.data.frame(cbind(p.CI.3yr$HOSP_ID, HG.solution$cluster))
names(HG.names) <- c("MIDB", "HG")


##########################################################################
##                                                                      ##
##  Rename the Hospital Groups                                          ##
##                                                                      ##
##########################################################################


#### Note:  The K-means + Ward's names clusters using random
####        numbers. This section will re-enumerate the Hospital
####        Groups based on an existing larger regional group.
####        (HSA - Health Service Area) and the sum of the beds
####        in the Hospital Groups. This section can be omitted
####        if re-enumerating is not necessary.
```

```
####        This sections also requires that the hospital
####        information file (hosp.info) has columns named
####        "HSA" and "BEDS".


# Attach initial cluster number to hospital information table
hosp.info <- merge(hosp.info, HG.names, by="MIDB", all.x=TRUE)

# Convert cluster number column to character format
hosp.info$HG <- as.character(hosp.info$HG)

# If hospitals were removed becasue they didn not have patient
# records, assign them to "NG"
hosp.info$HG[is.na(hosp.info$HG)] <- "NG"

# For each Hospital Group, find the HSA where the max number of
# hospitals falls inside. These lines of code assumes that there is
# a column named "HSA" in the hospital information table (hosp.info)
HG.HSA <- NULL
for (hg in 1:n.clusters) {
  sub <- hosp.info$HSA[hosp.info$HG == hg]
  t.sub <- table(sub)
  HG.HSA <- c(HG.HSA, names(t.sub[t.sub == max(t.sub)]))
}

###############################################
## Rename Hospital Groups by HSA and bed count
###############################################

# Make holder
HG.NEW <- NULL

# Make counter variable. Will hold the "last" Hospital Group
# name assigned
max.hg <- 0

# Get number of "regions" (HSAs)
hsa.list <- as.numeric(sort(unique(HG.HSA)))

# Start looping through the regions
for (hsa in hsa.list) {

  # Get Hospital Groups in region
  hsa.hgs <- which(HG.HSA == hsa)

  # Subset hospital information file to only hospitals
  # in these HSAs
  sub.hosp.info <- hosp.info[hosp.info$HG %in% hsa.hgs,]

  # Aggregate the number of hospital beds in each
  # Hospital Group. Assumes there is a column in hosp.info
  # named "BEDS"
  bed.totals <- aggregate(sub.hosp.info$BEDS, by=list(HG = sub.hosp.info$HG), sum)

  # Reorder aggregated table by total number of beds
  bed.totals <- bed.totals[order(bed.totals$x, decreasing=TRUE), ]

  # Change column type to character
  bed.totals$HG <- as.character(bed.totals$HG)

  # Make numbers for first and last Hospital Group in
```

```
  # this subset. Uses counter.
  f.hg <- max.hg+1
  l.hg <- nrow(bed.totals)+max.hg

  # Make join table
  j.HG.names <- as.data.frame(cbind(bed.totals$HG, f.hg:l.hg))

  # Name columns
  names(j.HG.names) <- c("HG_O", "HG_N")

  # Append the holder table
  HG.NEW <- rbind(HG.NEW, j.HG.names)

  # Advance counter
  max.hg <- l.hg

}

################################################################
## Attach new Hospital Group names to hospital information table
################################################################

hosp.info <- merge(hosp.info, HG.NEW, by.x="HG", by.y="HG_O", all.x=TRUE)

# Remove old cluster numbers
hosp.info$HG <- NULL

# Rename Hospital Group column
col <- which(names(hosp.info) == "HG_N")
names(hosp.info)[col] <- "HG"


#########################################################################
##                                                                     ##
##  Assign a new hospital to existing Hospital Groups                  ##
##                                                                     ##
#########################################################################


#### Note:  This code requires a 1 x n vector of hospital distances
####        to assign a hospital to the existing Hospital Groups
####        based on location. Uses a Euclidean distance measure from
####        the new hospital to the existing cluster centers.

###################################################
## Get original cluster centers from K-means object
###################################################

HG.centers <- as.data.frame(HG.solution$centers)

# Subset to only "travel distance" attributes
HG.centers <- HG.centers[,n.zip:ncol(HG.centers)]

# Attach new cluster names to cluster centers
HG.centers$HG_O <- rownames(HG.centers)
HG.centers <- merge(HG.centers, HG.NEW, by="HG_O")

# Remove old names and re-sort data
HG.centers$HG_O <- NULL
rownames(HG.centers) <- HG.centers$HG_N)
HG.centers$HG_N <- NULL
HG.centers <- HG.centers[order(as.numeric(rownames(HG.centers))),]
```

```
###################################
## Get new hospital or observation
###################################

# Get travel distance for new observation
new.hosp.loc <- read.csv("inputdata/new.hospital.location.csv")

# Remove characters from column names
# R adds an "X" to the column names that
# are only numeric values.
# Assumes hospital name is 4 characters long.
fix.names <- which(nchar(names(new.hosp.loc)) > 4)
names(new.hosp.loc)[fix.names] <- substr(as.character(names(new.hosp.loc)[fix.names]), 2,
      5)

# Test that columns match in new hospital and Hospital
# Group cluster centers
if (sum(names(new.hosp.loc) != names(HG.centers)) > 0) print("Columns do not match")

# Divide travel distances by the maximum travel distance
# between any hospitals in Michigan
new.hosp.loc <- new.hosp.loc / max

#####################################################
## Create function to measure Euclidean distance in
## n-dimensional space
#####################################################

euc.dist <- function(x1, x2) {
  dist <- sqrt(sum((x1-x2)^2))
  return(dist)
}

#######################################################
## Measure distance from new location to all existing
## Hospital Group centers
#######################################################

new.dists <- apply(HG.centers, 1, euc.dist, x2=new.hosp.loc)

# Get closest Hospital Group
HG.new.hosp.loc <- names(new.dists)[new.dists == min(new.dists)]

#### Note:  This is the Hospital Group that the new
####        hospital is assigned to

print(HG.new.hosp.loc)
```
